# Plant organellar signalling—back and forth and intertwined with cellular signalling

**DOI:** 10.1093/jxb/erac383

**Published:** 2022-11-19

**Authors:** Markus Teige, Matt Jones, Gabriela Toledo-Ortiz

**Affiliations:** Department of Functional & Evolutionary Ecology, University of Vienna, Djerassiplatz 1, 1030 Vienna, Austria; School of Molecular Biosciences, University of Glasgow, Glasgow G12 8QQ, UK; Lancaster University, Lancaster Enviroment Centre, Lancaster LA1 4YQ, UK

**Keywords:** Apocarotenoid, chloroplast, environmental signals, haem, mitochondria, photoreceptors, phytohormones, plastid development, retrograde, tetrapyrrole

**First observations that organelles are able to transmit their developmental and functional status back to the nucleus, where the majority of their proteins are encoded, date back almost half a century when impaired plastid protein synthesis in the *albostrians* barley mutant was found to also affect cytoplasmic protein synthesis (Bradbeer *et al.*, 1979). Later on, it was similarly described how mutations in the mitochondrial genome modulate the expression of nuclear-encoded genes in yeast (Parikh *et al.*, 1987). The term ‘retrograde organellar signalling’ was coined for these signals, and subsequently different genetic screens uncovered more and more players in these signalling pathways, mostly based on advanced genetic screens after chemical perturbation of plastid processes in plants (Woodson and Chory, 2008) or by employing yeast genetics for mitochondrial retrograde signalling (Liu and Butow, 2006).**

Subsequently, functional genetics studies over decades uncovered the chemical nature of some of those signals. These include ‘classical’ retrograde signalling metabolites such as tetrapyrrole intermediates or haem that are linked to chloroplast development and are therefore classified as ‘biogenic’ control signals (reviewed in Pogson *et al.*, 2008; Woodson and Chory, 2008), as well as ‘operational’ signals from the plastid (Pogson *et al.*, 2008) that build a cellular communication network decoding the languages of the chloroplast (Chan *et al.*, 2016). From these findings, the view emerged that organelles are functionally intertwined with other signalling pathways such as Ca^2+^ signalling, protein kinase signalling, or hormones during development and stress responses (Kmiecik *et al.*, 2016).

Over the past years, these connections were further elaborated, and the articles assembled in this Special Issue highlight various aspects of this integration of different signalling pathways. Starting with the fundamental question of ‘how it all began’, Khan and Van Aken (2022) performed a phylogenetic analysis and propose that the colonization of land was a likely driving force for the evolution of mitochondrial retrograde signalling in plants, while additional fine-tuning appeared in seed plants or even later. Such mechanisms for regulating chloroplast development are then reviewed by Liebers *et al.* (2022), hinting at functional feedback between plastid and cytosolic protein homeostasis in plastid retrograde signalling during chloroplast biogenesis. They suggest that dually localized nucleo-plastidic proteins coordinate chloroplast biogenesis with light-dependent control of seedling development. The role of light signals mediated by plant photoreceptors and their signalling components in chloroplastic anterograde and retrograde communication is reviewed by Griffin and Toledo-Ortiz (2022). In their review, they point out that phytochrome and cryptochrome photoreceptors are essential for tuning photomorphogenesis and chloroplast functions. However, their integration in the interorganellar communication cascades for proper environmental responsiveness is just beginning to be addressed. Importantly, too much light can pose a severe stress for chloroplasts and leads to the formation of carotenoid derivatives acting as retrograde chloroplast signals (Ramel *et al.*, 2012). Although this phenomenon has been described for a decade, novel players are still entering the stage, as summarized by Sierra *et al.* (2022). They highlight that apocarotenoid retrograde signals are additional highly complex components underpinning the link of the plastid metabolic status to plant development and environmental stress response.

The intimate links between chloroplast function and cellular stress responses are further discussed in another two reviews. Mackenzie and Mullineaux (2022) describe the concept of ‘sensory plastids’ in epidermal and vascular parenchyma cells. They point out that these plastids display shared features of dynamic morphology, proteome composition, and plastid–nuclear interaction to facilitate environmental sensing and signalling, which may suggest that such specializations within plastid populations align with different cellular properties. These could include primary and secondary metabolism, plant growth, organ development, and environmental sensing. In general, such regulatory processes in plant development and stress responses are regulated by different plant phytohormones, and their metabolism is tightly linked with chloroplasts. This concept is also discussed in the review by Bittner *et al.* (2022), who describe how plastids and mitochondria control various aspects of phytohormone signalling and host important steps of hormone precursor biosynthesis. These steps work alongside the contributions of plant hormones to organellar functions.

Altogether, we think that these new aspects in organellar signalling will provide important inputs for future research to understand the complex cellular networks involved in plant development and physiology, and particularly in view of improving stress resilience in response to environmental fluctuations.
